# *Legionella pneumophila* Persists within Biofilms Formed by *Klebsiella pneumoniae, Flavobacterium sp*., and *Pseudomonas fluorescens* under Dynamic Flow Conditions

**DOI:** 10.1371/journal.pone.0050560

**Published:** 2012-11-21

**Authors:** Catherine R. Stewart, Viraj Muthye, Nicholas P. Cianciotto

**Affiliations:** Department of Microbiology and Immunology, Northwestern University Medical School, Chicago, Illinois, United States of America; University of São Paulo, Brazil

## Abstract

*Legionella pneumophila*, the agent of Legionnaires' disease pneumonia, is transmitted to humans following the inhalation of contaminated water droplets. In aquatic systems, *L. pneumophila* survives much of time within multi-organismal biofilms. Therefore, we examined the ability of *L. pneumophila* (clinical isolate 130b) to persist within biofilms formed by various types of aquatic bacteria, using a bioreactor with flow, steel surfaces, and low-nutrient conditions. *L. pneumophila* was able to intercalate into and persist within a biofilm formed by *Klebsiella pneumoniae*, *Flavobacterium* sp. or *Pseudomonas fluorescens*. The levels of *L. pneumophila* within these biofilms were as much as 4×10^4^ CFU per cm^2^ of steel coupon and lasted for at least 12 days. These data document that *K. pneumoniae, Flavobacterium* sp., and *P. fluorescens* can promote the presence of *L. pneumophila* in dynamic biofilms. In contrast to these results, *L. pneumophila* 130b did not persist within a biofilm formed by *Pseudomonas aeruginosa*, confirming that some bacteria are permissive for *Legionella* colonization whereas others are antagonistic. In addition to colonizing certain mono-species biofilms, *L. pneumophila* 130b persisted within a two-species biofilm formed by *K. pneumoniae* and *Flavobacterium* sp. Interestingly, the legionellae were also able to colonize a two-species biofilm formed by *K. pneumoniae* and *P. aeruginosa*, demonstrating that a species that is permissive for *L. pneumophila* can override the inhibitory effect(s) of a non-permissive species.

## Introduction

The aquatic bacterium *Legionella pneumophila* is the agent of Legionnaires' disease, a serious form of pneumonia that is occurring with increasing incidence [1]–[4]. *L. pneumophila* is ubiquitous in natural and man-made water systems, and infection can occur following the inhalation of *L. pneumophila*-containing droplets produced by a variety of devices [5]. The widespread distribution of *L. pneumophila* results from the bacterium's ability to flourish within multiple types of niches, including survival in the planktonic phase, infection of and replication within protozoan hosts, and persistence within multi-organismal biofilms that cover surfaces within water systems [6]–[9]. With the goal of developing strategies for minimizing disease transmission [10], investigators have been utilizing laboratory models to understand when and how *L. pneumophila* is able to exist within biofilms. The chemical and physical parameters that influence the behavior of the legionellae in biofilms include the properties of the surface, the flow rate and turbulence of the liquid over the surface, the ambient temperature, carbon and metal concentrations, and the presence of biocides [11]–[22]. The first biological parameter that influences the bacterium's impact in biofilms is the presence of protozoa that are permissive for intracellular growth of legionellae. Indeed, various types of amoebae, including *Hartmannella vermiformis* and *Acanthamoebae castellanii*, greatly promote the growth of *L. pneumophila* within biofilms [21], [23]–[25]. Some studies have further concluded that replication within the biofilm requires the presence of protozoan hosts [23]–[26]. However, others have argued that *L. pneumophila* can grow in the absence of amoebal hosts by utilizing the matrix and nutrients provided by other bacteria within the biofilm [27]–[30]. Thus, the second critical biological parameter that influences the presence of *L. pneumophila* within biofilms is the type of bacterial species that inhabit the biofilm. At the very least, these organisms provide the matrix to which *L. pneumophila* can attach and persist prior to encountering an amoebal host. Although many studies have used microbial consortia, both defined and undefined, obtained from water systems to establish and study *Legionella*-containing biofilms in the laboratory [14], [22], [24], [26], [31]–[34], little is known about the relationships between *L. pneumophila* and particular bacterial species within the context of a dynamic biofilm. In the course of documenting the importance of amoebae for biofilms, Murga et al found that *L. pneumophila* (strain RI243) barely persisted (i.e., ≤10 CFU/steel coupon that provided 2.5 cm^2^ of surface area) within a multi-species biofilm composed of *Klebsiella pneumoniae*, *Pseudomonas aeruginosa*, and *Flavobacterium* sp. [24]. Using the same “CDC bioreactor” and steel coupons as Murga et al did, a later study found that a different strain of *L. pneumophila* (i.e., clinical isolate 130b) persisted in the *Klebsiella-Pseudomonas-Flavobacterium* biofilm at a level of 100–1000 CFU/coupon for a period of 15 days [35]. These data suggested that one or more these heterologous bacteria are capable of providing a biofilm that is conducive to the long-term persistence of *L. pneumophila*. Utilizing the same steel coupons as the previous two studies and the CDC bioreactor, which assesses bacterial colonization on surfaces in the presence of significant flow and in the absence of planktonic replication, we now demonstrate that *L. pneumophila* is able to persist at high levels (e.g., 10^4^–10^5^ CFU/coupon) when in a biofilm that is formed by just *K. pneumoniae*, *Flavobacterium* sp., or *Pseudomonas fluorescens* but not *P. aeruginosa*.

## Materials and Methods

### Bacterial strains and growth media

*L. pneumophila* 130b (ATCC strain BAA-74), also known as AA100 or Wadsworth, served as our wild-type strain [36]. Mutants of 130b that were examined included the *flaA* mutant NU347 which lacks flagella, *pilQ* mutant NU278 which lacks type IV pili, and *bbcB* mutant NU388 which lacks surfactant [36]–[38]. In order to help distinguish 130b and its derivatives from other bacteria in the biofilms, the chloramphenicol-resistant vector pMMB2002 [37] was placed into the strains. Legionellae were routinely grown at 37°C in buffered yeast extract broth or on buffered charcoal yeast extract (BCYE) agar [36]. The heterologous bacteria that were used to create biofilms were *Klebsiella pneumoniae* strain DMDS 92-08-28a, *Flavobacterium sp*. strain CDC-65, *Pseudomonas aeruginosa* ATCC strain 7700, and *Pseudomonas fluorescens* ATCC strain 17569 [24], [39]. These organisms can be found in potable-water environments, along with a wide variety of other bacteria and in some instances along with *L. pneumophila* [24], [40]–[44]. The bacteria were maintained on R2A media, which consists of, per liter, 0.5 g each of yeast extract, (Becton Dickinson [BD], Franklin Park, NJ), bacto-peptone (BD), bacto-tryptone (BD), and glucose, 0.39 g K_2_PO_3_·3H_2_O, 0.3 g sodium pyruvate, and 0.05 g MgSO_4_· 7H_2_O, pH 7.2 [45]. R2A agar consisted of R2A media plus 0.5 g/l soluble starch and 15 g/l agar. Unless otherwise noted, chemicals were purchased from Sigma-Aldrich (St. Louis, MO).

### Biofilm reactor

**Figure 1:**
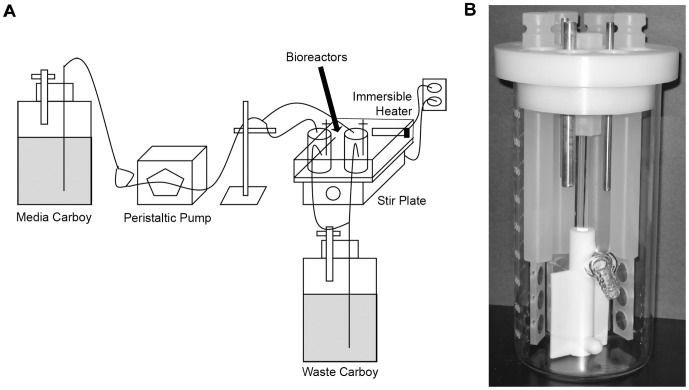
Schematic of the bioreactor system. (A) Representation of system set-up. Medium was pumped from the media carboy to the reactors by using a peristaltic pump. The bioreactors themselves were in a water bath that was maintained at 30°C by the use of an immersible aquarium heater. The bioreactors were kept spinning at a constant rpm over the course of the experiment. Liquid exited the bioreactors by gravity into a waste carboy. (B) Close-up view of the CDC biofilm reactor. The bioreactor has openings for 8 polypropylene rods. Each rod (four shown here, for clarity) contains three spaces for stainless steel coupons which can be removed to assay for bacterial CFU. At the center of the bioreactor is a stirring baffle that maintains constant shear stress. There is a spout located about 1/3 the length of the vessel from the bottom to allow for the exit of media.

In order to assess the ability of *L. pneumophila* to exist in biofilms, we utilized the CDC biofilm reactor (BioSurface Technologies, Bozeman, MT) [15], [46]. The bioreactor consisted of a 1-liter glass beaker with 8 polypropylene rods suspended from a ported lid. Each rod held 3 circular, 1.25-cm diameter stainless steel 316L coupons (BioSurface Tech.) that were positioned perpendicularly to a rotating baffle. Thus, the exposed surface area for each two-sided coupon was 2.5 cm^2^. Stainless steel 316L is a low-carbon version of 316 steel, a chromium-nickel stainless steel containing molybdenum (AK Steel Co., West Chester, OH). Such material is commonly used for cooling towers and plumbing materials, and previous studies have shown that *L. pneumophila* can exist in biofilms formed on stainless steel [13], [15], [22], [24], [35]. A schematic of the bioreactor set-up appears in Fig. 1.

### Biofilm experiments

On day-0, the bioreactor was inoculated with bacteria (i.e., 10^5^ CFU of *K. pneumoniae*, 10^8^ CFU of *Flavobacterium sp*., 10^8^ of CFU *P. aeruginosa*, and/or 10^8^ CFU of *P. fluorescens*) resuspended in 300 ml of R2A medium. The bioreactor was kept in static “batch” mode for 3 days at 30°C, 200 rpm [15]. On that third day, 10^10^ CFU of *L. pneumophila* were added to the bioreactor, and after 2 hours to allow for *Legionella* adherence to the biofilm, 1∶100 R2A (i.e., 1% R2A solution [vol/vol]) began to be pumped through the reactor at a rate of 1–2 ml/min. The bioreactors were run under continuous flow of 1∶100 R2A (30°C, 200 rpm) for up to an additional 12–14 days with samples being taken every 2–4 days. For sampling, one rod was removed and replaced with a sterile, blank rod. The coupons taken from the rod were washed twice in Butterfield buffer (42.5 mg/l KH_2_PO_4_) and then aseptically transferred to a 15-ml conical tube with 10 ml of Butterfield buffer for disaggregation. Each coupon was treated with 3 cycles of 30-sec sonication (Branson Sonifer 450D, 15% amplitude) followed by 1.5 min of vortexing [35]. Serial dilutions of the resulting suspension were then plated in triplicate on the appropriate media for enumerating bacterial CFU, resulting in a limit of detection equal to 10 CFU per coupon (i.e., 4 CFU/cm^2^). BCYE agar containing 100 U of polymyxin B and 6 mg/l chloramphenicol were used to assess *L. pneumophila* counts. To eliminate *P. fluorescens* from the sample, a heating step of 50°C for 30 min was required. Because *L. pneumophila* does not grow in R2A medium, the recovery of *L. pneumophila* CFU was a reflection of the organism's ability to attach to and persist in the biofilm. To enumerate the other species of bacteria, the sample was plated onto R2A agar. When *K. pneumoniae* and *Flavobacterium* sp. were used in the same reactor, 6 mg/l chloramphenicol was added to the R2A agar in order to assess *Flavobacterium* CFU.

## Results

### *L. pneumophila* colonizes and persists within monospecies biofilms of *K. pneumoniae* and *Flavobacterium sp*

**Figure 2:**
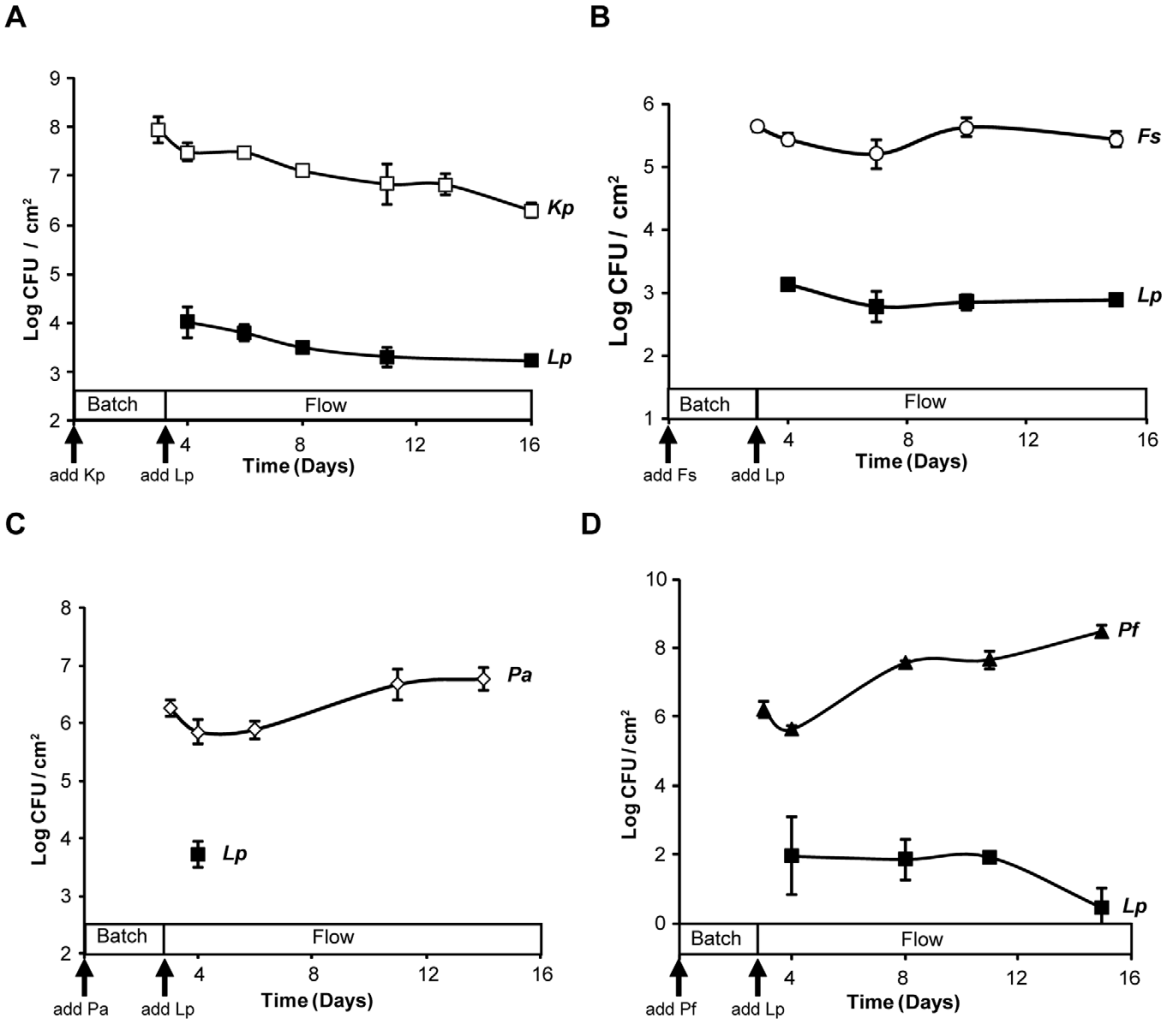
Persistence of *L. pneumophila* in monospecies biofilms formed by *K. pneumoniae, Flavobacterium sp., P. aeruginosa*, or *P. fluorescens*. A base biofilm of either *K. pneumoniae* strain DMDS 92-08-28a (□) (A), *Flavobacterium* sp. strain CDC-65 (Δ) (B), *P. aeruginosa* ATCC strain 7700 (◊) (C), or *P. fluorescens* ATCC strain 17569 (Δ) (D) pre-formed on stainless steel coupons was exposed to *L. pneumophila* strain 130b (▪) on day 3 and flow of 1∶100 R2A began at 1–2 ml/min. Each data point represents the averages and standard deviations of CFU obtained from the coupons within a single rod. The experiments shown are representative of four experiments for (A) and at least two for (B–D). In a repeat of the experiment using *P. aeruginosa* base biofilms, no legionellae were recovered at the initial sampling point.

To begin to determine if *L. pneumophila* can adhere to and persist within biofilms that had been formed by heterologous bacteria, we inoculated our bioreactors with *K. pneumoniae* or *Flavobacterium* sp. and then later added strain *L. pneumophila* strain 130b. By three days post-inoculation and prior to the introduction of *L. pneumophila*, *K. pneumoniae* heavily attached to the steel coupons, achieving levels between 4×10^7^ to 4×10^8^ CFU per cm^2^ (Fig. 2A). On the third day, 10^10^ CFU of *L. pneumophila* were added to the reactor and the flow of liquid through the system was established. Over the next 12 days of the experiment, the numbers of *K. pneumoniae* on the coupons declined gradually but at no point did the level fall below 4×10^6^ CFU per cm^2^ (Fig. 2A). Most importantly, *L. pneumophila* colonized the *Klebsiella* biofilm at ≥4×10^3^ CFU per cm^2^ and then persisted at this level throughout the time course. In the next series of experiments, *Flavobacterium* sp. CDC-65 was found to be capable of establishing a biofilm on the steel coupons albeit not as robustly as the *K. pneumoniae* strain had done (Fig. 2B). *L. pneumophila* strain 130b effectively intercalated into the *Flavobacterium* biofilm and was maintained over the entire course of the experiment at approximately 4×10^3^ CFU per cm^2^ (Fig. 2B). In two additional experiments, when 10^10^ CFU of *L. pneumophila* strain 130b were added to the bioreactor in the absence of any other bacteria, no CFU were recovered from the coupons (limit of detection  = 10 CFU) indicating that *L. pneumophila* cannot establish its own biofilm in this model system. In summary, *K. pneumoniae* and *Flavobacterium* were both able to provide a biofilm environment that is conducive to colonization and high-level persistence by *L. pneumophila*.

### *L. pneumophila* colonizes and persists within monospecies biofilms of *P. fluorescens* but not *P. aeruginosa*

We next sought to determine whether *P. aeruginosa* could provide a suitable base biofilm for *L. pneumophila* colonization and persistence. When the base biofilm consisted of only *P. aeruginosa*, initial attachment of *L. pneumophila* was often observed and at a level (approximately 3×10^3^ CFU/cm^2^) that was similar to what had been seen with base biofilms consisting of *Flavobacterium sp*. or *K. pneumoniae* (Fig. 2C). However, no *L. pneumophila* CFU were recovered two days later or at subsequent time points. These data suggest that *P. aeruginosa* produces a factor(s) that prevents the maintenance of strain 130b. Furthermore, they indicate that the persistence of *L. pneumophila* that we had observed when using the *K. pneumoniae* and *Flavobacterium* sp. biofilms was not simply an artifact of the bioreactor system or our protocol. To determine if the result obtained with *P. aeruginosa* was typical for *Pseudomonas* species, we performed an experiment using *P. fluorescens*, another bacterium that is often found in water samples alongside *L. pneumophila* [40]. When the base biofilm consisted of only *P. fluorescens*, *L. pneumophila* was able to both intercalate and persist (Fig. 2D), albeit at a level that was approximately 10-fold less than we had observed with the *Klebsiella* or *Flavobacterium* species. Thus, *L. pneumophila* strain 130b was able to colonize and persist within dynamic biofilms formed by some but not all species of *Pseudomonas*.

### *L. pneumophila* persists within various two-species biofilms

**Figure 3:**
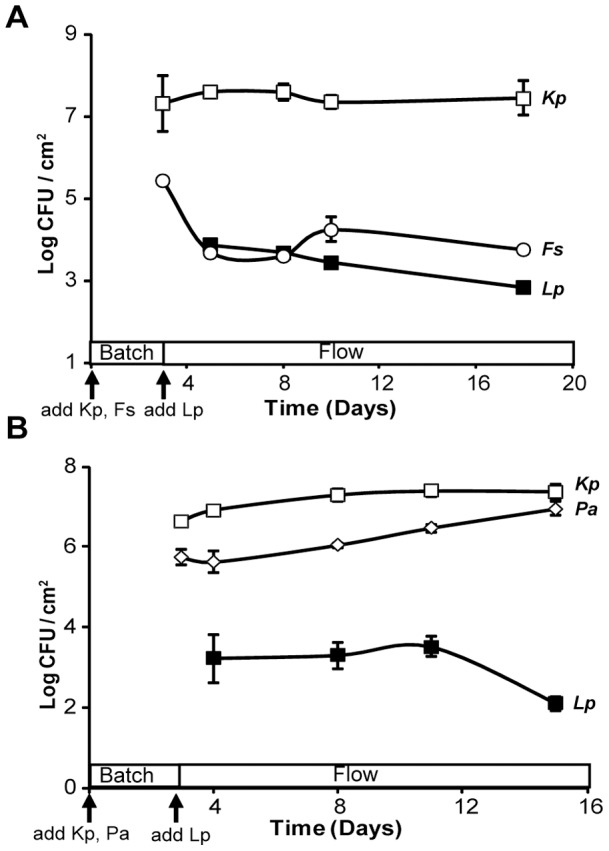
Persistence of *L. pneumophila* in two-species biofilms formed by *K. pneumoniae* and *Flavobacterium* sp. or *K. pneumoniae* and *P. aeruginosa*. A base biofilm of either *K. pneumoniae* strain DMDS 92-08-28a (□) and *Flavobacterium sp*. strain CDC-65 (◊) (A) or *K. pneumoniae* strain DMDS 92-08-28a (□) and *P. aeruginosa* ATCC strain 7700 (◊) (B) pre-formed on stainless steel coupons was inoculated with *L. pneumophila* strain 130b (▪) on day 3 and flow of 1∶100 R2A began at 1–2 ml/min. Each data point represents the averages and standard deviations of colony counts from a single rod, and the experiments shown here are representative of two experiments.

Given the ability of *L. pneumophila* to integrate into a monospecies biofilm of *Klebsiella* or *Flavobacterium*, we next sought to determine how strain 130b would fare when exposed to a biofilm consisting of both *K. pneumoniae* and *Flavobacterium* sp. On day-3 and prior to the addition of legionellae, the *Klebsiella* and *Flavobacterium* organisms demonstrated an ability to co-exist within the biofilm formed on steel coupons, with each achieving levels that were comparable to what had been observed in the monospecies experiments (Fig. 3A). More importantly, *L. pneumophila* integrated into the biofilm and persisted over the remaining 12-day course of the experiment. The presence of legionellae within the multi-species biofilm was maintained between 400 to 4000 CFU per cm^2^, which was comparable to that of the flavobacteria (Fig. 3A) and also similar to the degree to which the legionellae persisted when in combination with *Klebsiella* alone or *Flavobacterium* alone (Figs. 2A and 2B). Taken together, these data indicate that *L. pneumophila* is capable of persisting relatively well within biofilms that contain multiple heterologous species and that *K. pneumoniae* and *Flavobacterium* sp. alone or in combination are not inhibitory to *L. pneumophila*. When strain 130b was exposed to a multi-species biofilm consisting of *K. pneumoniae* and *P. aeruginosa*, the legionellae colonized and persisted at approximately 4000 CFU per cm^2^ for at least 8 days and then dropped in numbers about 10-fold by the end of the experiment (Fig. 3B). The decline in *L. pneumophila* appeared coincident with increasing numbers of *P. aeruginosa* in the biofilm. These data confirm that *L. pneumophila* can persist within different sorts of multi-species biofilms. Furthermore, they indicate that the dramatic inhibitory effect of *P. aeruginosa* on *L. pneumophila* that we had observed in earlier experiments was absent or greatly reduced when a third species (e.g., *K. pneumoniae*) is present in the mixed biofilm.

### *L. pneumophila* flagella, pili, and surfactant are not required for attachment to or persistence in a dynamic biofilm formed by *K. pneumoniae*

**Figure 4:**
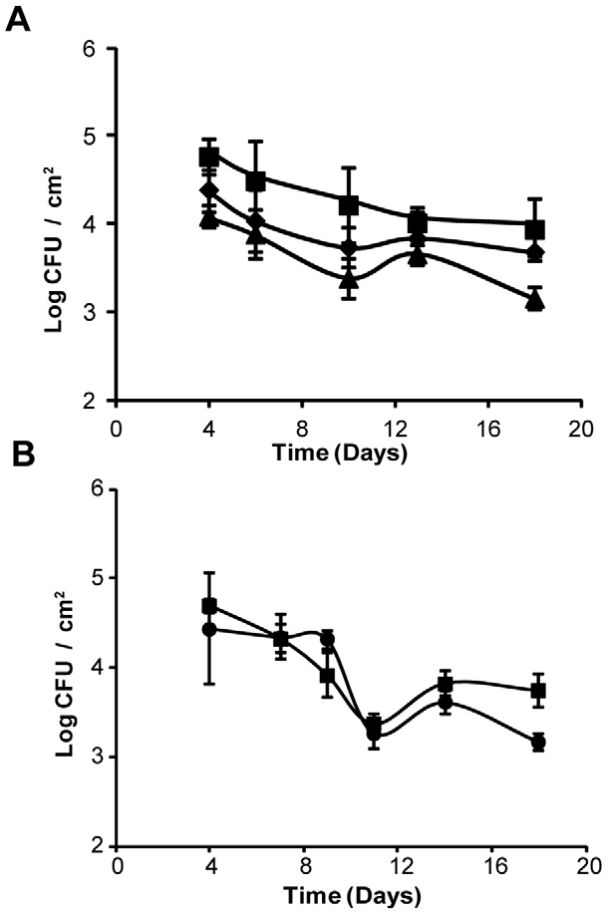
Persistence of *L. pneumophila* mutants in a K. *pneumoniae* biofilm. A base biofilm of *K. pneumoniae* strain DMDS 92-08-28a pre-formed on stainless steel coupons was inoculated in (A) with either *L. pneumophila* wild-type strain 130b (▪), *flaA* mutant NU347(pMMB2002) (♦), or *pilQ* mutant NU278(pMMB2002) (▴) and in (B) with either *L. pneumophila* wild-type strain 130b (▪) or *bbcB* mutant NU388 (•) on day 3 and flow of 1∶100 R2A began at 1–2 ml/min. Each data point represents the averages and standard deviations of colony counts from a single rod, and the experiments presented are representative of two repeats.

*L. pneumophila* is known to exhibit swimming via the action of a polar flagellum [47], twitching motility and adhesiveness that are associated with type IV pili [48]–[50], and sliding motility and anti-microbial activity that are dependent on a secreted biosurfactant [36], [38]. Because factors such as these are implicated in biofilm formation by other bacteria [7], [38], we separately tested flagella, pili, and surfactant mutants of strain 130b in a bioreactor that had been previously inoculated with *K. pneumoniae*. In each of the experiments using a mutant, the numbers of *K. pneumoniae* on the steel coupons were comparable to what we had observed when testing parental strain 130b (data not shown), indicating that *Legionella* flagella, pili, and surfactant do not significantly impact the behavior of the heterotroph. In the first experimental set-up which consisted of three reactors running in parallel, colonization and persistence by the flagella and pilus mutants mirrored that of the wild-type strain; i.e., all displayed approximately 4×10^3^–4×10^4^ CFU per cm^2^ over the entire time course (Fig. 4A). Although one or both of the mutants appeared to be less prominent compared to wild-type at several time points, these differences were not statistically significant. In the next round of experiments which consisted of two reactors running in parallel, the surfactant mutant behaved as wild-type did in terms of both colonization and persistence (Fig. 4B). In summary, these data indicate that flagella, type IV pili, and surfactant are not required for the ability of *L. pneumophila* to colonize and persist within a base biofilm consisting of *K. pneumoniae*.

## Discussion

Our data demonstrate, for the first time, that *L. pneumophila* is able to intercalate into and persist within a monospecies biofilm formed by *K. pneumoniae*, *Flavobacterium* sp. or *P. fluorescens*. *K. pneumoniae* (strain DMDS 92-08-28a) and *Flavobacterium* sp. (strain CDC-65) were comparable in terms of their ability to support *L. pneumophila* persistence, while *P. fluorescens* (ATCC strain 17569) was slightly less permissive. Importantly, our experiments used a dynamic bioreactor with flow conditions, steel surfaces, and low-nutrient media. Furthermore, the persistence that we observed reached as much as 4×10^4^ CFU per cm^2^ and lasted at least 12 days. Currently, it is not possible to discern whether the bacterial load on the steel coupons reflects bacteria that persist but do not replicate or is the sum of growth plus loss due to the flow within the bioreactor; e.g., it is possible that, at some time point(s), the legionellae can replicate in 1∶100 R2A medium that has been “conditioned” by some of the biofilm communities.

Only one previous study examined the ability of *L. pneumophila* to engage a monospecies biofilm formed by *K. pneumoniae*, but the investigators found that the bacterium was not able to attach or persist [21]. The possible reasons for our differing results include differences in the *Legionella* strains used (130b vs. JR32), the *Klebsiella* strains (DMDS 92-08-28a vs. 21UHC), the substrata (i.e., steel vs. glass and plastic), the media, the flow rates and other physical aspects of the dynamic model system. Thus, it would appear that the ability of *L. pneumophila* to intercalate and persist within a *K. pneumoniae* biofilm is a variable trait. No previous study using a dynamic biofilm model examined the ability of *L. pneumophila* to attach to and persist within a monospecies biofilm formed by *P. fluorescens*, although one prior study found that *P. fluorescens* strain SSD (but not *P. fluorescens* ATCC 49838) inhibited the ability of *L. pneumophila* (strain “Lp-1”) to adhere to the wells of a polystyrene microtiter plate [42]. To our knowledge, no previous study using any sort of biofilm model examined the ability of *L. pneumophila* to attach to and persist in a monospecies biofilm made by *Flavobacterium* sp. Further review of the literature revealed that there are only a few other examples of a heterologous bacterium supporting the presence of *L. pneumophila* in biofilms. Using dynamic flow models, Mampel et al and Vervaeren et al found that biofilms formed by *Empedobacter breve*, *Microbacterium* sp., or *Pseudomonas putida* are able to provide a base that is conducive to *L. pneumophila* long-term persistence [21], [51]. Another study found that *L. pneumophila* intercalates into a dynamic biofilm formed by *Sphingomonas* sp. and can be isolated again 12 hours later; however, because no other time points were examined, it is difficult to conclude whether these data represent *L. pneumophila* persistence [52]. Because these past studies did not report CFU and used different protocols, it is not possible to rank *K. pneumoniae, Flavobacterium* sp., *P. fluorescens*, *E*. *breve*, *Microbacterium* sp., and *P. putida* (and *Sphingomona*s sp.) in terms of their capacity to support *L. pneumophila* persistence within dynamic biofilms. Finally, it is worthwhile to add that other past studies have reported that *Acinetobacter lwoffii* and *Mycobacterium chelonae* individually promote biofilm formation by *L. pneumophila* under static conditions; i.e., by assessing bacterial numbers bound to either the wells of a plastic microtiter plate or PVC coupons placed into the wells [42], [53].

In contrast to the results obtained with *K. pneumoniae*, *Flavobacterium* sp., and *P. fluorescens*, we observed that a monospecies biofilm formed by *P. aeruginosa* ATCC strain 7700 was not conducive to the persistence of *L. pneumophila* strain 130b. In some of our experiments, the legionellae initially associated with the biofilm but were lost within two days of further incubation, suggesting that *L. pneumophila* can attach to a matrix produced by *P. aeruginosa* but is unable to resist inhibitory substances and/or effectively compete for space or nutrients. Although the reason why *P. aeruginosa* impedes the persistence of *L. pneumophila* in our system remains to be determined, one possible speculation derives from the fact that purified homoserine lactones produced by *P. aeruginosa* inhibit another strain of *L. pneumophila* in a static (microtiter-plate) biofilm assay [54]. To our knowledge, only one previous study examined the interaction of *L. pneumophila* with a monospecies biofilm of *P. aeruginosa* under dynamic flow conditions; in that case, *L. pneumophila* strain JR32 never attached to the biofilm that had been formed by *P. aeruginosa* strain K [21]. In considering our result with those of this previous study, it would appear that *P. aeruginosa* biofilms can be unsuitable for *L. pneumophila* for a variety of reasons. *P. aeruginosa* is not alone in its refractory effect on *L. pneumophila*. Using a dynamic biofilm model, Mampel et al found that *L. pneumophila* JR32 was unable to attach to a biofilm formed by *Corynebacterium glutamicum* and unable to persist within a biofilm made by *Acinetobacter baumannii* [21]. Examining static biofilms in microtiter-plates, others found that *L. pneumophila* (i.e., strain Lp-1 and NCTC 12821) can be inhibited by *Aeromonas hydrophila*, *Burkholderia cepacia*, *Acidovorax* sp., and *Sphingomonas* sp. [42], [53].

In light of the ability of *L. pneumophila* to persist in a monospecies biofilms formed by either *K. pneumoniae* or *Flavobacterium* sp., it was not surprising that the organism also persisted well in a mixed biofilm consisting of both *K. pneumoniae* and *Flavobacterium* sp. Given this result and the fact that *L. pneumophila* persisted in a biofilm formed by *P. fluorescens*, we strongly suspect that *L. pneumophila* would intercalate into and persist within a two-species biofilm consisting of either *K. pneumoniae* and *P. fluorescens* or *Flavobacterium* sp. and *P. fluorescens*. An interesting and arguably more surprising result was the observation that *L. pneumophila* persisted relatively well within in a mixed biofilm formed by *K. pneumoniae* and *P. aeruginosa*, whereas *L. pneumophila* was completely unable to persist in a biofilm formed by *P. aeruginosa* alone. This experiment documented, for the first time, that the permissiveness of one species (e.g., *K. pneumoniae*) for *L. pneumophila* can be dominant over the non-permissiveness of another species (e.g., *P. aeruginosa*) for *L. pneumophila*. It will be interesting, in the future, to investigate how *K. pneumoniae* is able to erase the negative effect(s) of *P. aeruginosa*. Our data also help explain why *L. pneumophila* was able to persist to some degree in dynamic biofilms that consisted of *K. pneumoniae*, *Flavobacterium* sp., and *P. aeruginosa* [24], [35]; i.e., *K. pneumoniae* and *Flavobacterium* sp. likely provided factors that directly stimulated the persistence of *L. pneumophila* while at the same time dampening the inhibitory effect(s) of *P. aeruginosa*. In light of these results, it might be instructive to “deconstruct” the other past studies that had exposed *L. pneumophila* to biofilms consisting of a different combination of known bacteria. For example, several studies showed that *L. pneumophila*, though not replicating, could persist within a four-species biofilm that was composed of *A. hydrophila*, *Escherichia coli*, *Flavobacterium breve*, and *P. aeruginosa* [25], [55]. Another study found that a seven-species biofilm consisting of *A. baumannii, C. glutamicum, E. breve, K. pneumoniae, Microbacterium sp., P. aeruginosa*, and *P. putida* was not even conducive to persistence [21].

At this point, it is not clear how *K. pneumoniae*, *Flavobacterium* sp., and *P. fluorescens* are facilitating the integration and persistence of *L. pneumophila* within their biofilms. Since strains of these species/genera produce capsular, extracellular matrix material [56]–[58], it is likely that they are providing an appropriate substrate for the attachment of *L. pneumophila*. Additionally, they may be directly or indirectly providing nutrients that promote the survival and/or growth of the legionellae. Another important question is what factors encoded by *L. pneumophila* promote the organism's ability to attach and persist within the biofilms formed by these other bacteria under dynamic flow conditions. Using a set of specific *L. pneumophila* 130b mutants and a model biofilm derived from *K. pneumoniae*, we could not uncover a role for flagella, type IV pili, or surfactant. The lack of a role for flagella in attachment and persistence could be a reflection of the fact that our system provided a sufficient mechanism for bringing the legionellae into contact with the biofilm on the steel coupons. The lack of a required role for type IV pili suggests that *L. pneumophila* strain 130b has other surface molecules that mediate attachment to the *K. pneumoniae* biofilm. Previously, Lucas et al found that another type IV pilus mutant of *L. pneumophila* had a modestly reduced ability to colonize a mixed biofilm consisting of *K. pneumoniae, Flavobacterium* sp., and *P. aeruginosa* [35]. In trying to reconcile our results with this past study, it would appear that *L. pneumophila* attachment to the mixed biofilm is a combination of events, including non-type IV pilus-mediated attachment to that portion of the biofilm consisting of *K. pneumoniae* and/or *K. pneumoniae* matrix, and type IV pilus-mediated attachment to other portions that are not dominated by *K. pneumoniae*. Although our study and that of Lucas et al are the only ones to have investigated any *L. pneumophila* mutants in a mixed biofilm model with dynamic flow conditions, several groups have obtained results using just legionellae in the static, microtiter plate-based assay. Factors that have been identified as being important by that measure include the FliA sigma factor, a collagen-like adhesin, twin-arginine translocation, and nitric-oxide sensors [21], [59]–[62]. Factors that were not found to be important in the static biofilm model include flagella, type IV pili, Dot/Icm type IV secretion system, the Lvh type IV secretion system, the Lqs quorum-sensing system, and the regulatory factors, RpoS, LetA, and CsrA [21], [63]. In sum, very little is known about the factors encoded by *L. pneumophila* that mediate its attachment to and persistence within biofilms created by other bacteria. We posit that the mechanisms of *L. pneumophila* survival within natural biofilms are likely to be quite variable, depending upon the types of bacteria that constitute the biofilm base as well as the presence of different protozoan hosts and non-microbial environmental factors.
